# Supplementary material for: Development of an asthma policy model for Canada: Lifetime Exposures and Asthma outcomes Projection
Source: PLoS One. 2026 May 13;21(5):e0348878. doi: 10.1371/journal.pone.0348878 (PMC13170856; doi:10.1371/journal.pone.0348878)
Supplement: S1 File — (DOCX) [file pone.0348878.s001.docx]

Supplementary Materials

**Table of Contents**

[1. Demographics module 1](#_Toc223814216)

[2. Trends in antibiotic prescription rates among infants 4](#_Toc223814217)

[3. Calibration of the asthma incidence and prevalence equations 6](#_Toc223814218)

[3.1 Estimation of crude asthma prevalence and incidence 6](#_Toc223814219)

[3.2 Asthma reassessment 10](#_Toc223814220)

[3.3 Incorporating the effect of risk factors 11](#_Toc223814221)

[4. Exacerbation 16](#_Toc223814222)

[4.1 Annual exacerbation rate for each level of asthma control 16](#_Toc223814223)

[4.2 Exacerbation severity 16](#_Toc223814224)

[4.3 Initialisation of exacerbations 17](#_Toc223814225)

[4.4 Calibration of exacerbations 17](#_Toc223814226)

[5. Operationalising the microsimulation 18](#_Toc223814227)

[6. Internal validation results 22](#_Toc223814228)

[References 29](#_Toc223814229)

# 1. Demographics module

Statistics Canada provides population projections under 9 different scenarios.[1] The demographics module was calibrated to Statistics Canada’s M3 population projection as we found that, among the scenarios, this scenario resulted in the lowest root mean squared error of the total population at the national level when compared with the observed data in 2020 and 2021 (**Supplementary Table 1)**.

| **Population projection scenario** | **Root mean squared error** |
| --- | --- |
| Medium growth 3 | 71708 |
| Medium growth 2 | 71714 |
| Medium growth 1 | 71714 |
| Medium growth 4 | 71714 |
| Medium growth 5 | 71741 |
| Slow aging | 102175 |
| High growth | 122869 |
| Fast aging | 132307 |
| Low growth | 152424 |

**Supplementary Table 1.** Root mean squared error in the population projection by scenario.

Birth is one of the two ways for individuals to enter the simulation after the initialisation of the population. The number of births by sex and year was based on the estimate (2000-2019) or projection (2020-2065) from Statistics Canada.[1,2] Statistics Canada does not provide a breakdown of immigrants and emigrants by sex and age in their population estimates and projections. We decided to model the net immigrants and net emigrants via model calibration. For each year, we calculated the number of individuals required to immigrate and emigrate, by sex and age, to match the estimated or projected size of the population. The corresponding proportions for the different combinations of sex and age yielded empirical distributions for immigration and emigration. We then used these empirical distributions to assign sex and age of each immigrant (via a categorical distribution) and to determine whether an individual emigrates (via a Bernoulli distribution), respectively.

For mortality, we used the estimated life tables to model whether an individual dies at the end of each time cycle.[3] In the current version, the mortality rate was not differentiated between individuals with asthma and those without asthma, as deaths due to asthma are very rare[4]. In Canada, the proportion of all-cause deaths due to asthma annually between 2000 and 2020 never exceeded 0.14%[5]. To reflect projected increase in life expectancy, we modified the latest life table (2020) for each sex by calibrating the probability of death across all ages as follows:

$$logit\left( p\left( sex,age,year \right) \right)=logit\left( p\left( sex,age,2020 \right) \right)-\beta_{sex}\left( year-2020 \right) (Eq. 1)$$

where $p\left( sex,age,2020 \right)$ is the probability of death from the 2020 life table. Determination of the calibration constant $\beta_{sex}$ was based on life expectancy as Statistics Canada provides only projected life expectancy of an individual at birth in year 2068 for each of its projection scenarios, not projected life tables.

Given the probability of death for each sex and age in 2020 and a value for $\beta_{sex}$, the probability of death for each sex and age in 2068 given by Equation 1 allows evaluation of the corresponding projected life expectancy at birth (our target values are 87.0 years for males and 90.1 years for females). The life expectancy in year $t$ is calculated based on the current life table for year $t$, following the notations and definitions in Strauss et al.[6]. Let $I\left( x \right)$ be the number of persons alive at age $x$, $d\left( x \right)=I\left( x \right)-I\left( x+1 \right)$ be the number of deaths in the interval $\left( x,x+1 \right)$ for persons alive at age $x$, $q\left( x \right)$ be the probability of dying at age $x$, $L\left( x \right)$ be the total number of person-years lived by the cohort from age $x$ to $x+1$, and $T\left( x \right)$ be the total number of person-years lived by the cohort from age $x$ until all members of the cohort have died (i.e., the sum of $L\left( x \right)$ from age $x$ to the maximum age, which is 110 years in the Canadian life table generated by Statistics Canada). Then $e\left( x \right)$, the remaining life expectancy of persons alive at age $x$, is calculated as $e\left( x \right)=T\left( x \right)/I\left( x \right)$.

Life expectancy at birth is $e\left( 0 \right)=T\left( 0 \right)/I\left( 0 \right)$. To calculate $T\left( 0 \right)$, $L\left( x \right)$ is needed for all ages $x$. Note that $L\left( x \right)$ is the sum of the years lived by the $I\left( x+1 \right)$ persons who survive the interval, and the $d\left( x \right)$ persons who died during the interval. The former contribute exactly 1 year each, while the latter contribute, on average, approximately half a year. For the boundary of ages of 0 and 110, a smaller contribution ($<0.5$) is usually used for the former, and a larger contribution (between 1 and 2) is usually used to for the latter (since they live longer than 110 years but not very much longer). In general, with $w\left( x \right)$ denoting the contribution made by members of the cohort who die at age $x$, $L\left( x \right)=I\left( x+1 \right)+w\left( x \right)d\left( x \right)$. We chose, by trial and error, $w\left( x \right)=0.2$ for $x=0$ , 1.4 for $x=110$, and 0.5 for all other values of $x$. This choice of $w\left( x \right)$ matched the calculation of the life expectancy at birth for the 2020 life table by Statistics Canada.

In summary, using Equation 1 and initial values of $\beta_{sex}$, we first calculated $q\left( x \right)$ for all ages $x$ in 2068. We used $I\left( 0 \right)=100,000$ (other values can be used as the value of $I\left( 0 \right)$ cancels out during evaluation of life expectancy) and calculated $d\left( x \right)=I\left( x \right)q\left( x \right)$ and $L\left( x \right)=I\left( x+1 \right)+w\left( x \right)d\left( x \right)$. Then we computed $T\left( x \right)$ and finally $e\left( x \right)$. We solved for $\beta_{sex}$ by minimising the absolute error between the projected and targeted life expectancy values using a bisection method (uniroot function in R).

# 2. Trends in antibiotic prescription rates among infants

We observed a decreasing trend of antibiotic exposure, with males receiving more courses of antibiotics than females. Extrapolation eventually leads to near zero rates of antibiotic exposure which is unrealistic. To prevent this unrealistic scenario, we assumed a minimum rate of 50 (per 1,000 persons) after consultation with the steering committee. We used this truncated rate parameter in the negative binomial model to simulate the number of antibiotic prescriptions in the first year of life for each virtual individual (**Supplementary Fig 1**). Further details of this analysis are provided elsewhere.[7]


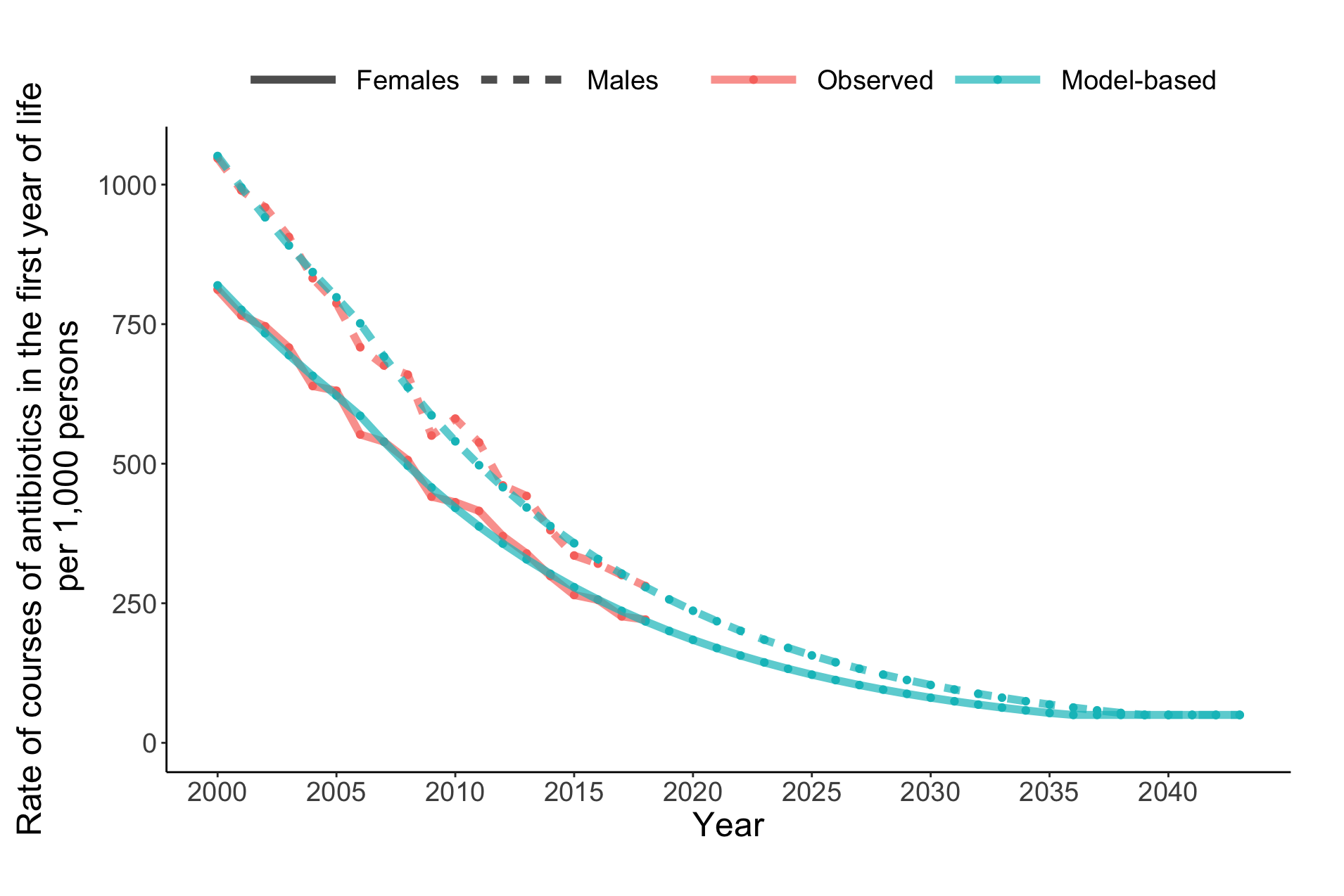


**Supplementary Fig 1.** Trends in the rate of courses of antibiotics in the first year of life by sex (solid: females, dashed: males, red: observed values in the population-based administrative databases of British Columbia, blue: model-based values).

# 3. Calibration of the asthma incidence and prevalence equations

## 3.1 Estimation of crude asthma prevalence and incidence

To obtain estimates of crude asthma prevalence and incidence at the national level, we used two data sources: the Canadian Community Health Survey (CCHS) and administrative databases of BC. CCHS is a cross-sectional self-reported survey for 12 years or older. It provides asthma prevalence estimates, based on self-report, at the national level but does not report on asthma prevalence for ages less than 12 years or on asthma incidence for any age.[8] On the other hand, both asthma prevalence and incidence rates could be estimated from the BC administrative data. However, in the administrative data, asthma labelling was made using the following case definition based on the diagnostic codes (ICD codes-10-CA: J45; ICD-9-CA: 493): one or more asthma-related hospitalisation, or two or more physician visits within one year, or one or more physician visits and two or more asthma prescriptions within one year (for a list of asthma prescriptions, see the BC Chronic Disease Registries Case Definitions[9]). This case definition has been independently validated against chart review.[10]

We found that prevalence did not differ much between BC and Canada in the CCHS data. However, there was a considerable discrepancy in asthma prevalence between the CCHS and BC administrative data (**Supplementary Fig 2**), reflecting different methods for asthma labelling. As CCHS was a self-reported survey which is subject to self-selection and response bias, we deemed the estimates from the BC administrative data to be more reliable. As such, we assumed that nationwide asthma prevalence and incidence rates were equal to those from the BC administrative data.


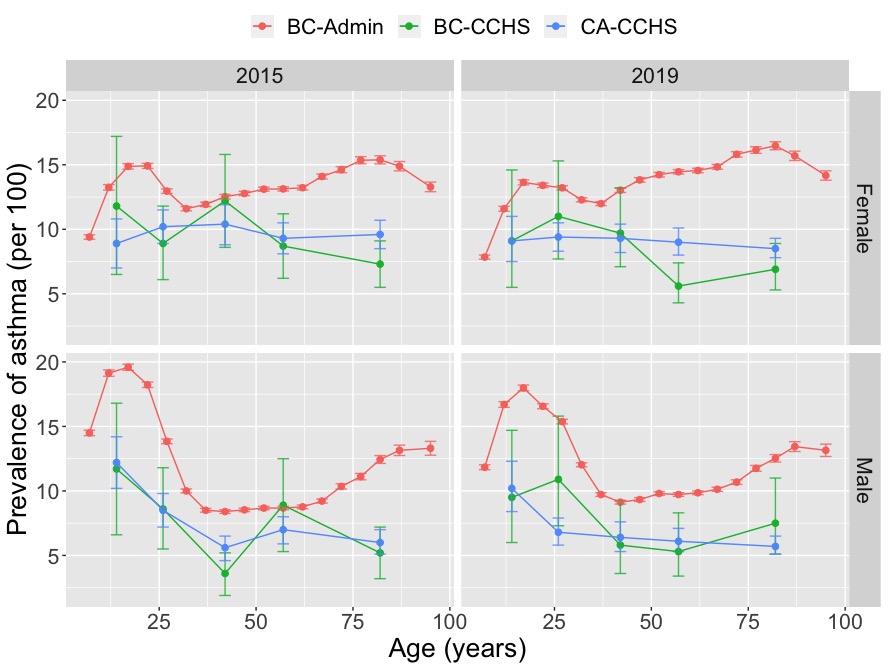


**Supplementary Fig 2.** Observed asthma prevalence across age in 2015 and 2019 for British Columbia (BC) and Canada (CA) based on the BC administrative data (red) and CCHS data (green: BC; blue: CA) by sex. CCHS: Canadian Community Health Survey

In the BC administrative data, we had information on observed prevalence and incidence from 2000 to 2019, stratified by 5-year age bands (0-4, 5-9, …, 84-89, 90+ years), biological sex, and calendar year.[11] To estimate the prevalence and incidence for each age bin, we first took the mid-point of the five-year age bands as the age for the corresponding prevalence and incidence. We discarded data on age $>$65 years due to potential mislabeling among older population and assumed that the incidence and prevalence rates for age $>$63 years (the mid-point of the last age band) remained constant at the rates for 63 years of age. For asthma incidence, we fitted a linear regression model with the log of the incidence rate as a linear combination of time, sex, poly(age,5) (recall that poly($x,q$) stands for the polynomials of $x$ up to degree $q$), as well as interaction terms of sex and poly(age,5) (**Supplementary Fig 3**). For asthma prevalence, we fitted a linear regression model with the log of the prevalence rate as a linear combination of poly(time,2), sex, poly(age,5) and all their interactions (**Supplementary Fig 4**).


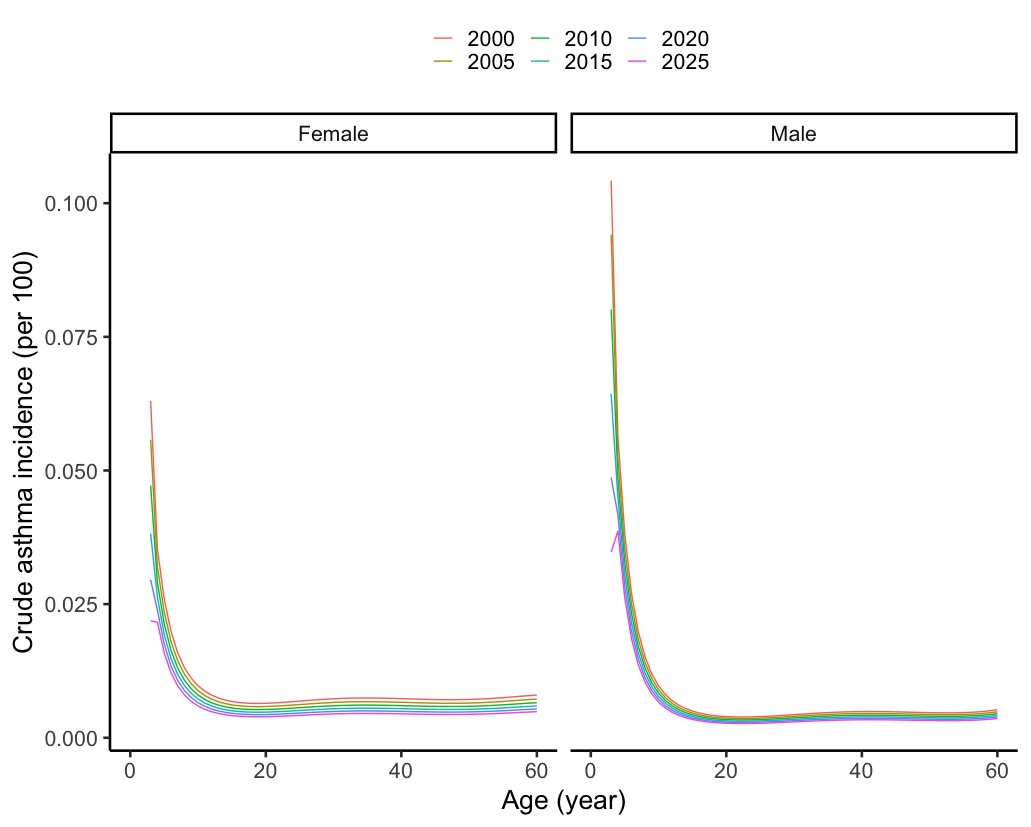


**Supplementary Fig 3*.*** Estimated asthma incidence for selected years by sex using the administrative data of British Columbia.


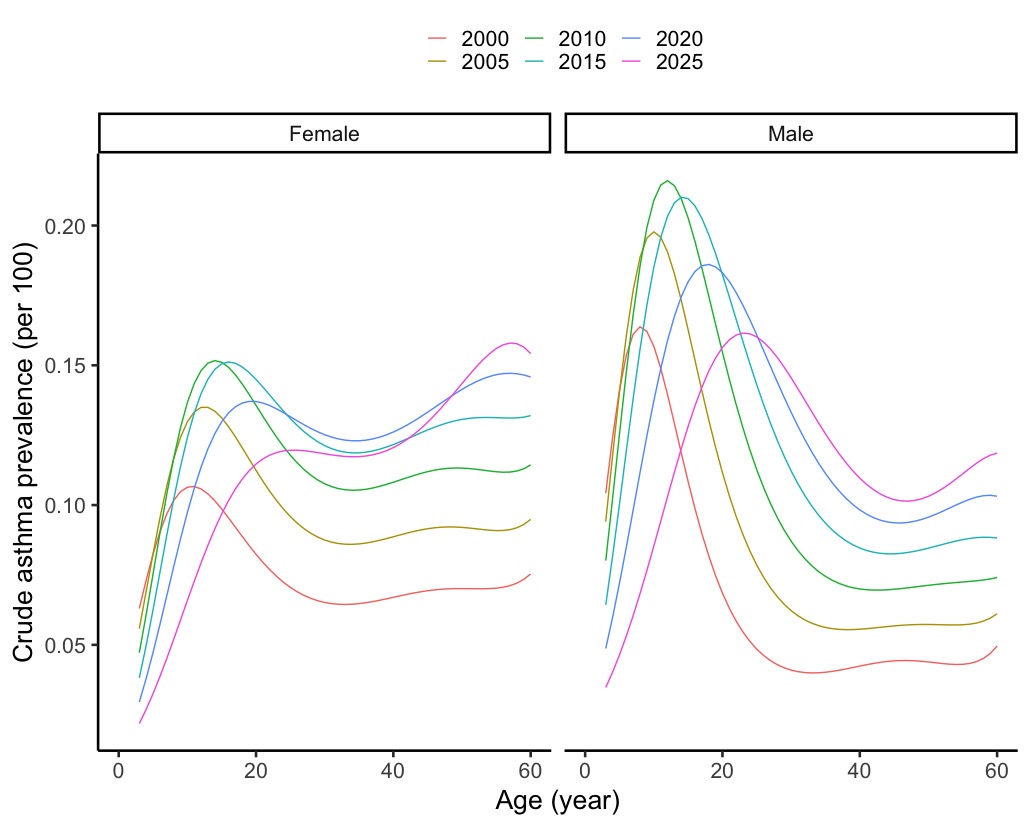


**Supplementary Fig 4*.*** Estimated asthma prevalence for selected years by sex using the administrative data of British Columbia.

For the past (prior to 2000), we assumed the estimates of prevalence and incidence from 2000. For the future (2020 onwards), we assumed that current incidence and prevalence trends continued up to 2025 and stayed constant thereafter. For the initial population and immigrants, the asthma prevalence estimates could be used to assign the asthma label to all individuals 3 or more years of age. For individuals not labelled as having asthma, the asthma incidence estimates (except for individuals less than 3 years of age) could be used to simulate whether an individual becomes labelled as having asthma in each time cycle. Of note, this implies we assume that immigrants have the same asthma incidence and prevalence rates as Canadians.

While these crude asthma prevalence and incidence estimates could be used to simulate asthma cases in the asthma model, the asthma model would generate excess asthma cases without accounting for asthma dormancy or remission (e.g., asthma prevalence rises from early childhood to young adulthood but then falls). In addition, we still need to incorporate risk factors. In the next section, we describe how we calibrated the crude asthma prevalence and incidence estimates through the asthma reassessment submodule before incorporating the risk factors.

## 3.2 Asthma reassessment

Asthma prevalence varies across age, characterised by a hump in early age.[12] Asthma is not curable and from a biologic perspective, its core pathology does not disappear.[13] Such variation is instead explained by the ‘dormancy’ of asthma, a prolonged period with no asthma symptoms. Such dormancy might be endogenous (e.g., outgrowing of asthma from childhood to adulthood) or exogenous (e.g., change in environment and risk of asthma triggers).[14] As well, in the community, misdiagnosis of asthma occurs mostly due to confounding by other allergic diseases or respiratory diseases,[15] which can also explain reduction in the prevalence of asthma by age. Correspondingly, we modelled the clinical status of asthma by a sub-module, asthma reassessment, to evaluate whether an individual labelled as having asthma remains as such for any time cycle.

We assume the following relationship among asthma incidence, prevalence, and reassessment. For specific sex and age, the number of individuals labelled as having asthma at the current year $t$ consists of individuals labelled as having asthma from the past year ($t-1$) who remain labeled as having asthma and individuals without an asthma label from the past year who become labelled as having asthma in the current year. For specific sex and age (arguments dropped for brevity), let $prev_{t}$ be the probability (or equivalently the proportion) of being labelled as having asthma in year $t$, $inc_{t}$ be the probability of becoming labelled as having asthma, and $p_{t}\left( reassessment \right)$ be the probability of maintaining the asthma label. Then the relationship can be described as follows:

$$\begin{matrix} prev_{t}= & prev_{t-1}p_{t}\left( reassessment \right)+\left( 1-prev_{t-1} \right)inc_{t} \end{matrix} \left( Eq. 2 \right)$$

We solved for $p_{t}\left( reassessment \right)$ for each year, sex, and age, as estimates of all the other quantities are available from the previous section. In the microsimulation, an individual is labelled as having asthma based on the asthma prevalence equation when they enter the simulation. In a subsequent time cycle, if they are labelled as having asthma, their label is reassessed and maintained with $p_{t}\left( reassessment \right)$. If not, their asthma label is determined by the asthma incidence equation.

## 3.3 Incorporating the effect of risk factors

In this subsection, we describe how the effect of the risk factors, namely family history of asthma at birth and infant (< 1 year of age) antibiotic exposure, are estimated and incorporated into the asthma incidence and prevalence equations.

Using the CHILD study data, Patrick et al.[16] found that family history of asthma was associated with an increase in the risk of being labelled as having asthma. They used a multivariable logistic regression to establish the association with the prevalence of asthma at the age of 3 years with an odds-ratio [OR] of 1.13 (95%CI: 0.66–1.95) and at the age of 5 years with an OR of 2.40 (95%CI: 1.13–5.09). We used linear interpolation on the log OR scale to estimate the risk at the age of 4 years, and for age greater than 5 years, we assumed that the risk stayed constant at the level for 5 years of age. That is, the log odds ratio for having family history of asthma at birth (for age $\geq$ 3 years) can be expressed as:

$$\log\left( OR \right)=\beta_{0}+\beta_{1}\left( \min\left( age,5 \right)-3 \right), \left( Eq. 3 \right)$$

where $\beta_{0}=log\left( 1.13 \right)$ and $\beta_{1}=\left( \log\left( 2.40 \right)-log\left( 1.13 \right) \right)/2.$ This equation corresponds to $\mathrm{FHA}_{j}\left( \cdot\right)$ (‘Effect of $\mathrm{FHA}$’) in **Table 1**.

A recent systematic review found support for the association between exposure to antibiotics in early life and the risk of being labelled as having asthma.[17] However, the association is mostly attributable to exposure to antibiotics in the first year of life.[18] In consultation with the steering committee, we assumed an association only for the first year of life. To obtain an estimate of the age-specific dose-response of the number of courses of antibiotics in the first year of life on the risk of being labelled as having asthma, Lee et al.[7] carried out a meta-analysis using the summarised data from the systematic review. Applying strict inclusion-exclusion criteria, they included six studies that reported on the dose response relationship with low risk of bias. They fitted a random-effects meta-regression model with the number of courses of antibiotics (0, 1, 2, 3, 4, 5 or more) and age of asthma diagnosis as covariates. They found a detrimental dosage effect up to 7 years of age and a diminishing effect with age of antibiotic exposure in the first year of life. We followed their assumption of no effect of the exposure to antibiotics in the first year on the risk of asthma beyond 7 years of age.

We made a further assumption to facilitate the calibration (discussed next). As the antibiotic prescription rate was not high, the probability of having more than three courses of antibiotics in the first year of life was practically zero. Hence, we reclassified the levels 3, 4, and 5+ together as one level, 3+, and the log OR for 3+ was assumed to be equal to the log OR for 3. That is, the log odds ratio for having any number of courses of antibiotics (dose) in the first year of life (for age $\in\{3,..,7\}$ years and dose $>$ 0) can be expressed as:

$$\log\left( OR \right)=\beta_{0}+\beta_{1}\left( age-3 \right)+\beta_{2}\min\left( dose,3 \right), \left( Eq. 4 \right)$$

where $\beta_{0}=1.826,$ $\beta_{1}=-0.225$, and $\beta_{2}=0.053.$ These values were based on the meta-analysis in a separate study mentioned earlier[7]. This equation corresponds to $\mathrm{IAE}_{j}\left( \cdot\right)$ (‘Effect of $\mathrm{IAE}$’) in **Table 1**.

Now incorporating the effect of risk factors into the asthma incidence and prevalence equations requires calibration, so that the marginal asthma prevalence (i.e., the sum of the products of the prevalence of each combination of the risk factors and the corresponding asthma prevalence) remains calibrated.

At calendar year $t$, sex $s$, and age $a$, let $X\left( t,s,a \right)=0,1,2,...,q$ be a categorical risk factor that takes levels $x=0,1,\ldots,q.$ For brevity, we drop the arguments $t,s,a$ in the following text. Let $p_{target}$ be the target marginal asthma prevalence, $OR_{x}$ be the association between the risk factor level $x$ and asthma labeling, and $p_{x}$ be the prevalence of the risk factor level. With $p_{prev}(x)$ denoting the asthma prevalence at level $x$ and $\beta_{0}^{target}$ denoting the logit of the target asthma prevalence, we seek a correction term $\delta$ for the intercept in the following asthma prevalence equation

$$logit\left( p_{prev}\left( x \right) \right)=\beta_{0}^{target}+log\left( OR_{x} \right)-\delta, \left( Eq. 5 \right)$$

such that the marginal asthma prevalence is calibrated (i.e., $p_{target}=\sum_{x} p_{x}p_{prev}\left( x \right)$) while maintaining the ORs. There is a unique solution to this optimisation problem (monotone decreasing in $\delta$), and we used the Broyden–Fletcher–Goldfarb–Shanno (BFGS) algorithm to solve for $\delta$. Multiple categorical risk factors can be represented as a single categorical risk factor, and the same method can be applied.

To obtain the calibrated asthma prevalence equation in **Table 1**, we took the following steps for each year, sex, and age. First, we represented the logit of the crude asthma prevalence by $\beta_{0}^{crude}+\vec{\beta}_{1}^{logit}\text{poly(year,2)}*\text{sex}*\text{poly(age,5)}$. These terms represent $\beta_{0}^{target}$ in Equation 5. The effect of level $x$ of the risk factors FHA and IAE is incorporated with $\mathrm{FHA}_{prev}$ and $\mathrm{IAE}_{prev}$, which represent $\log\left( OR_{x} \right)$ in Equation 5. Finally, we evaluated $\beta_{0}=\beta_{0}^{crude}-\delta$ by $\beta_{0}$ to obtain the final form of the calibrated equation.

Calibration for the asthma incidence equation is more complicated since the effects of the risk factors are unknown and need to be estimated such that both the marginal asthma incidence and the ORs in the asthma prevalence equation remain unchanged. We assumed that the same form of relationship between the risk factors and asthma prevalence for asthma incidence ($\mathrm{FHA}_{j}$ and $\mathrm{IAE}_{j}$ in **Table 1**) and that the risk factor equations for asthma prevalence and incidence are equal at age of 3 years (i.e., $\mathrm{FHA}_{inc}=\mathrm{IAE}_{inc}$ and $\mathrm{FHA}_{prev}=g_{prev}$ at age of 3 years). In other words, the values of $\beta_{0}$ in $\mathrm{FHA}_{inc}$, and $\beta_{0}$ and $\beta_{2}$ in $\mathrm{IAE}_{inc}$ are equal to those of $\mathrm{FHA}_{prev}$ and $\mathrm{IAE}_{prev}$, respectively. Our goal is then to optimise the $\beta_{1}$’s in $\mathrm{FHA}_{inc}$ and $\mathrm{IAE}_{inc}$.

Required inputs are the ORs for asthma prevalence, calibrated prevalence, and the probability distribution of the risk factor(s) from the previous time step, and odds ratios for asthma prevalence, crude incidence, and reassessment probabilities from the current time. We also need to specify initial values for the $\beta_{1}$’s (we used the corresponding values from $\mathrm{FHA}_{prev}$ and $\mathrm{IAE}_{prev}$). The first step is to compute, for each combination of sex and age, an odds ratio for being labelled as having asthma for each level $x$ of the risk factor relative to its reference level for the current time step. Put differently, we need a 2x2 contingency table of proportions, not counts, for that level $x$.

To construct this contingency table, we first need the contingency table of level $x$ from the previous time step. Given an odds ratio of level $x$, the proportion of individuals labelled as having asthma (among individuals in level $x$ and the reference level), and the proportion of individuals being at level $x$ from the previous time step, the table can be obtained using a method by Bonett.[19] Let $a_{0},b_{0},c_{0},d_{0}$ represent this table, with $a_{0}$ and $c_{0}$ being the proportion of individuals not labelled as having asthma at the reference level and at level $x$, and $b_{0}$ and $d_{0}$ being the proportion of individuals labelled as having asthma at the reference level and at level $x$.

Specifying initial values for the ORs in the incidence equation followed by calibration for the overall incidence rate provides a revised incidence equation. With the incidence rate for the reference level ($inc_{0}$) and level $x$ ($inc_{x}$), we now construct the corresponding contingency table for the current time step. The first cell $a$ is equal to the proportion of individuals at the reference level who either lose their asthma label $\left( b_{0}\left( 1-p\left( reassessment \right) \right) \right)$ or are not newly labelled as having asthma $\left( a_{0}\left( 1-inc_{0} \right) \right)$. The second cell $b$ can be obtained by subtraction: $b=a_{0}+b_{0}-a$. The cells $c$ and $d$ are similarly obtained from $c_{0},d_{0},$ and $inc_{x}$. We then compute the OR for asthma prevalence, compare it to the target OR for level $x$ from the asthma prevalence equation, and record the absolute difference. To find a set of values of the $\beta_{1}$’s in the asthma incidence equation, we aggregated the absolute differences over all combinations of year (up to the stabilisation year, 2025), sex, and age, and used the iterative BFGS algorithm until the aggregated difference was less than ${10}^{-10}$.

# **4.** Exacerbation

## 4.1 Annual exacerbation rate for each level of asthma control

Based on the Economic Burden of Asthma (EBA) study,[20] we first obtained the annual rate of exacerbation, as 0.347/year, as well as the proportion of time spent in the control levels: well-controlled (WC) = 0.340, partially-controlled (PC) = 0.474, and uncontrolled (UC) = 0.186. An analysis of the GOAL study[21] provided the (rounded) annual exacerbation rates for each asthma control level: rate(WC) = 0.1, rate(PC) = 0.2, and rate(UC) = 0.3. Thus, we assumed that the annual exacerbation rate for an PC/UC asthmatic individual is twice/thrice higher than for a WC asthmatic individual. Using the annual exacerbation rate from the GOAL study, we solved for the conditional rates in the relationship, overall rate = P(WC) * rate(WC) + P(PC) * rate(PC) + P(UC) * rate(UC), yielding: rate(WC) = 0.188, rate(PC) = 0.376, rate(UC) = 0.564.

## 4.2 Exacerbation severity

Asthma exacerbations are commonly classified as mild, moderate, severe, or very severe (or life-threatening) retrospectively based on the level of healthcare utilisation required to treat them.[12,22] To assign the severity of exacerbation, we used data from the Symbicort Given as Needed in Mild Asthma II study, a double-blind multi-centre clinical trial with individuals with mild asthma (n=4,176).[23] The proportion of exacerbation by severity was 49.5% for mild, 19.5% for moderate, 28.3% for severe and 2.6% for very severe.[23,24] We used those values as the shape parameter for a Dirichlet distribution to generate a preliminary vector of probabilities for the severity levels. We then incorporated the effect of very severe exacerbations on subsequent events as estimated in another Canadian study.[25] If an individual labelled as having asthma had a history of very severe exacerbation, the probability for the very severe level increased (details are provided in the online table). The probabilities for the other levels were correspondingly scaled such that the sum of the probabilities was 1. Finally, given the total number of exacerbations for an individual labelled as having asthma in a year and the final probability vector for the severity levels, we generated the number of exacerbations in each severity level with a multinomial distribution.

## 4.3 Initialisation of exacerbations

We needed to assign whether any very severe exacerbations were previously experienced by each individual labelled as having asthma in a prevalent population. To do so, we first needed to determine the number of time cycles that individual was labelled as having asthma since asthma can be reversible. Calculating this was computationally burdensome due to an explosion of possible states. To simplify, we assumed that asthma was not reversible in these individuals and ran a mini-simulation to determine the time cycle the individual was labelled as having asthma and then calculated the probability of having no very severe exacerbations from that incidence to the present assuming independence across cycles. Finally, we tossed a coin to determine whether this individual had at least one very severe exacerbation. Our simplifying assumption implies the number of years that the individual was labelled as having asthma was overestimated. However, given the low chance of asthma reversibility in our dataset, we posited that overestimation was not too severe.

## 4.4 Calibration of exacerbations

We performed calibration for asthma exacerbation to match the rate of asthma-related hospitalisations, which are equivalent to very severe exacerbations, observed in Canada by year, sex, and age.[26] For each year, sex, and age, we computed the predicted proportion of time spent in each of the asthma control levels and then calculated the predicted annual rate of exacerbations. Under the simplifying assumption that individuals labelled as having asthma had no past history of very severe exacerbations, we calculated the predicted rate of very severe exacerbation per unit of general population. Subsequently, for each year, sex, and age, we compared it with the observed value to obtain a calibration multiplier value and the logarithm of the multiplier was added to the exacerbation equation as $\beta_{0}$. Of note, our assumption implies that calibration will result in slightly higher rates than observed.

# 5. Operationalising the microsimulation

Before we discuss the microsimulation at the operational level, we briefly explain our rationale for implementing the model in Julia and Python. The model was initially programmed in Julia, as Julia offers the flexibility of an interpretative language (e.g., R and Python) and the near-efficiency of a low-level compiled language (e.g., C++), making it a powerful tool for computationally intensive calculations without using a low-level language.[27] Despite these attractive features, Julia does not yet have the popularity of Python or R. As such, we implemented a Python version as well.

Now we describe how the structural equations in each of the five main modules were constructed and used to simulate individual-level characteristics and asthma events in the microsimulation. This is communicated alongside pseudocode for the core of the microsimulation (**Supplementary Fig 5**).

The input parameters of the microsimulation (lines 0-4) determine the maximum age of each virtual individual examined (110 is the default), the starting year of the simulation, the period for which the simulation is run, and the size of the population (e.g., 10% of the whole population). Subsequently, the maximum calendar year is calculated (line 6), and the starting year is set to 2019 if it exceeds 2019 (lines 8-10) as explained in the demographics module. We also initialise an array to store events over all virtual individuals.

For each calendar year, we determine how many virtual individuals to simulate based on the demographics module (lines 16-20). If it is the starting year, we need to simulate the entire initial population. If not, we need to simulate newborns and immigrants. Then we simulate each virtual individual (line 22) and begin with initialising their risk factors (line 26). Sex and age are assigned based on the demographics module, and family history of asthma at birth and infant antibiotic exposure are simulated based on the risk factors module. Next, we determine their asthma attributes from the previous year (lines 28-38). This happens only if their age is greater than 3 years since asthma attributes are not given for individuals less than 3 years of age. We assign the asthma label based on the asthma prevalence equation and simulate asthma control and exacerbations if they are labelled as having asthma.

Next, the virtual individual enters a conditional loop (line 40). They exit the loop if they meet any of the conditions: they are dead or emigrate, their age exceeds the prespecified maximum age, or their current calendar year is greater than the prespecified maximum year. In each time cycle, we first run the asthma occurrence module to determine whether they are labelled as having asthma (lines 42-46). If they are labelled as having asthma, then we simulate asthma-related outcomes, namely asthma control and exacerbations (lines 48-53). Then we call the payoffs module to evaluate their utility and costs (line 55). We check whether they emigrate via the emigration submodule (line 57) or die via the mortality submodule (line 59). If they die or emigrate, we update their status so they will exit the loop (line 62). Otherwise, we increment their age and year by 1 (line 64).

After all the virtual individuals are simulated and exit the simulation across all the years, the simulation stops, and the outcomes matrix is returned to the user.


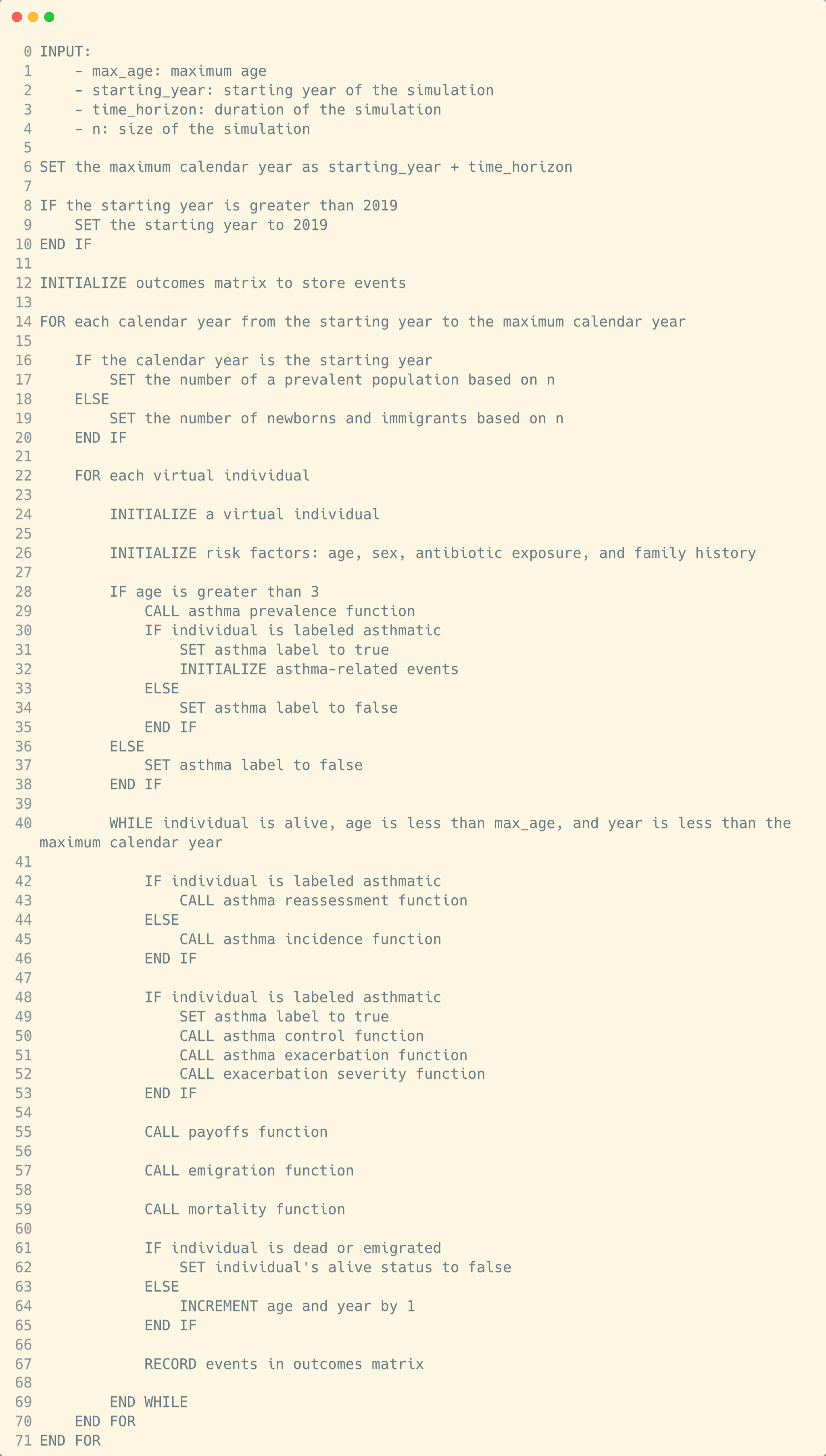


**Supplementary Fig 5.** Pseudocode of the of the Lifetime Exposures and Asthma outcomes Projection model.

# 6. Internal validation results


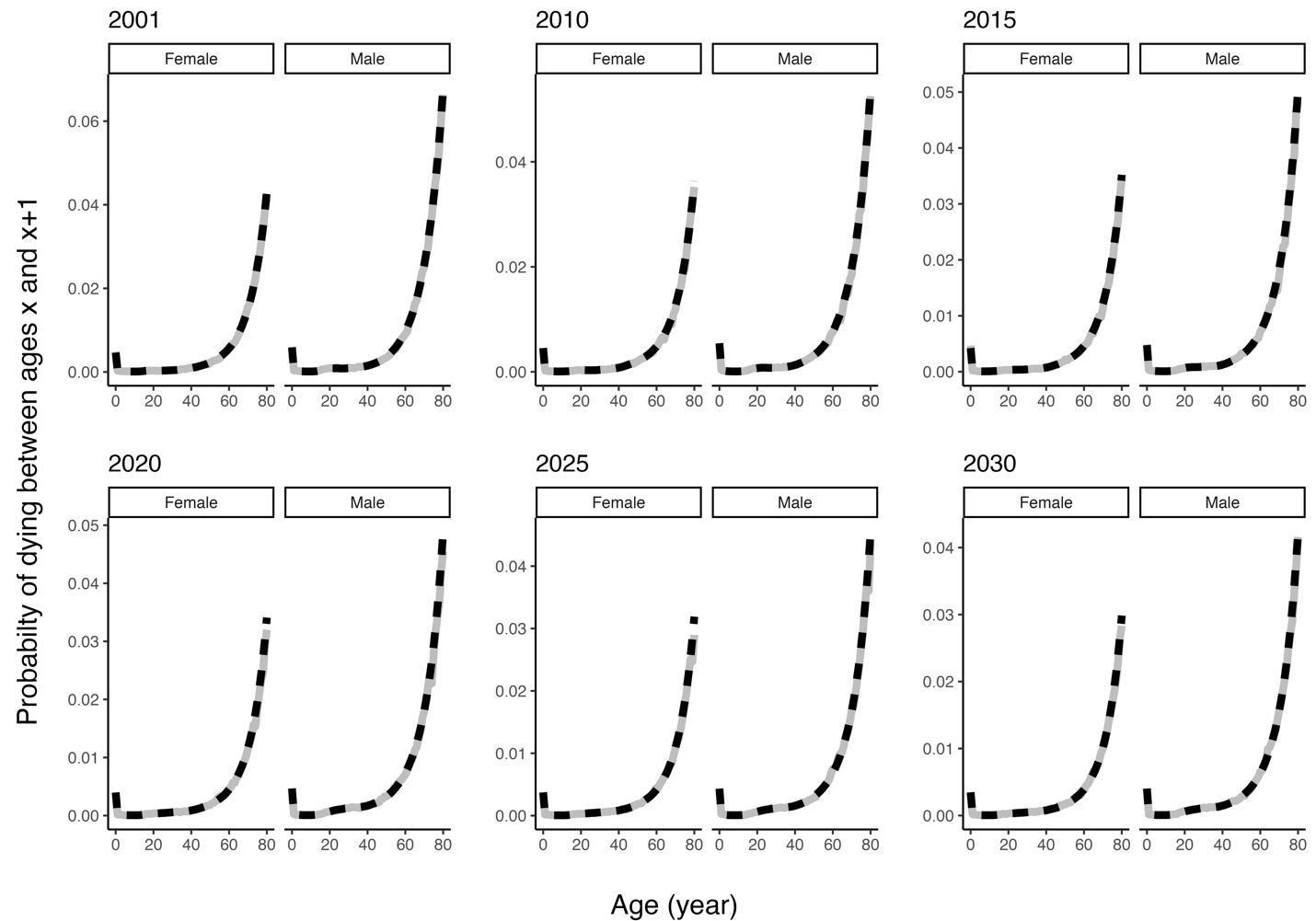
**Supplementary Fig 6.** Mortality by sex (left: males; right: females) for the model (grey solid) and Statistics Canada (black dashed).


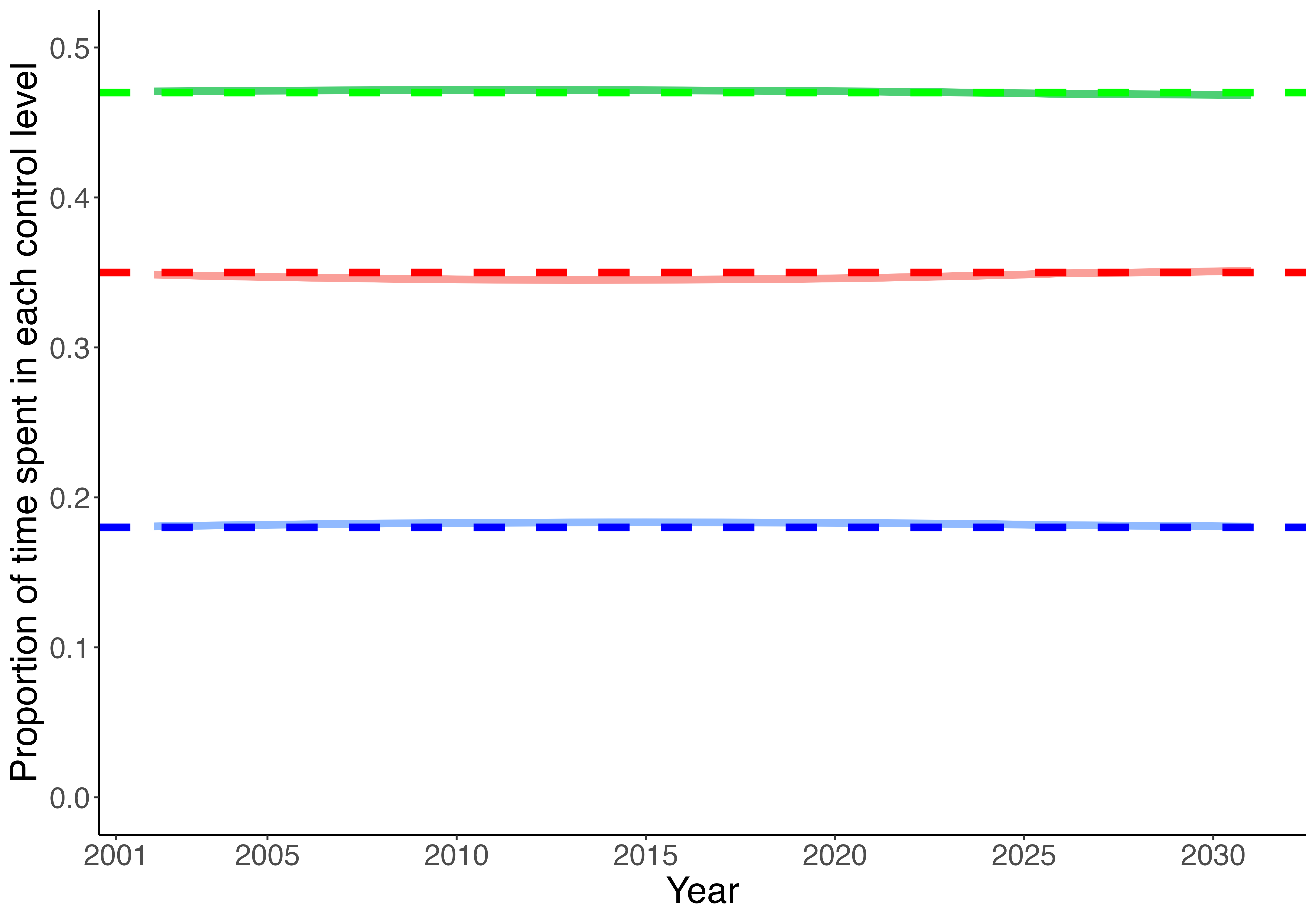
**Supplementary Fig 7.** Asthma control levels (red: well-controlled; green: partially controlled; blue: uncontrolled) by the model (solid) and target (dashed).


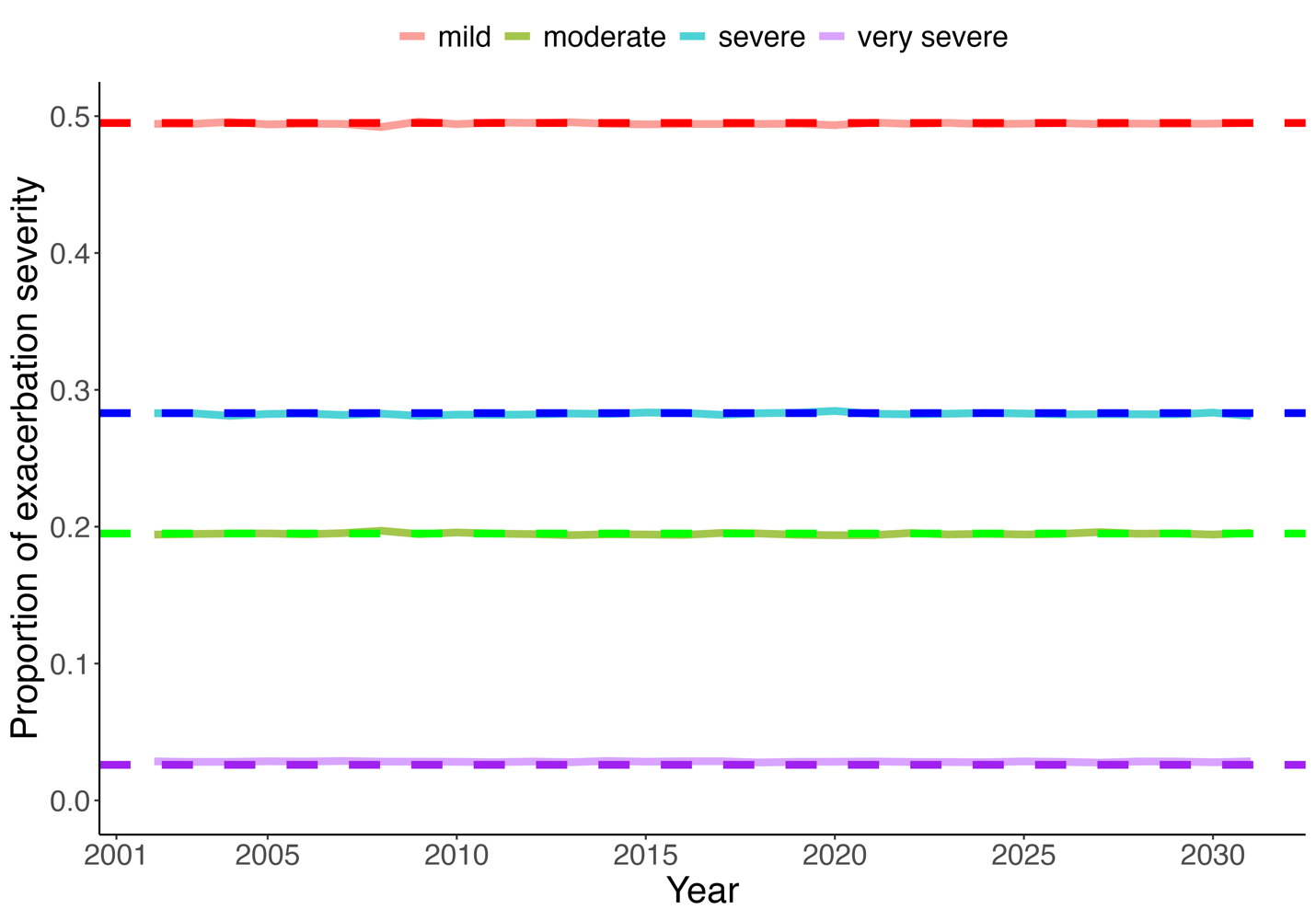
**Supplementary Fig 8.** Simulated (solid) and target (dotted) asthma exacerbation severity levels (red: mild; blue: moderate; green: severe; purple: very severe).


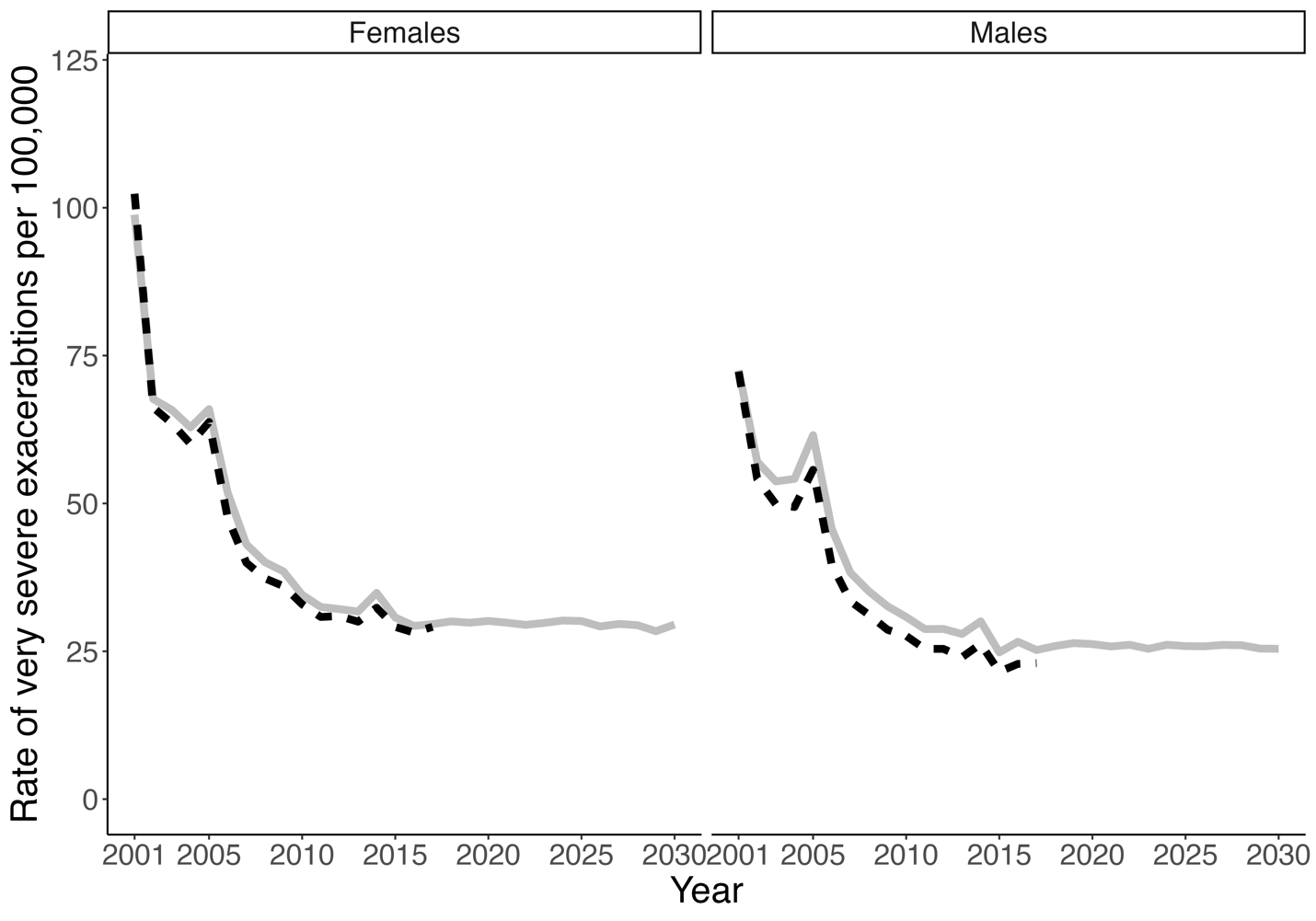
**Supplementary Fig 9.** Comparison of simulated (grey solid) and target (black dotted) very severe asthma exacerbations (asthma-related hospital admissions) per 100,000 general population by sex (left: females; right: males) across years.


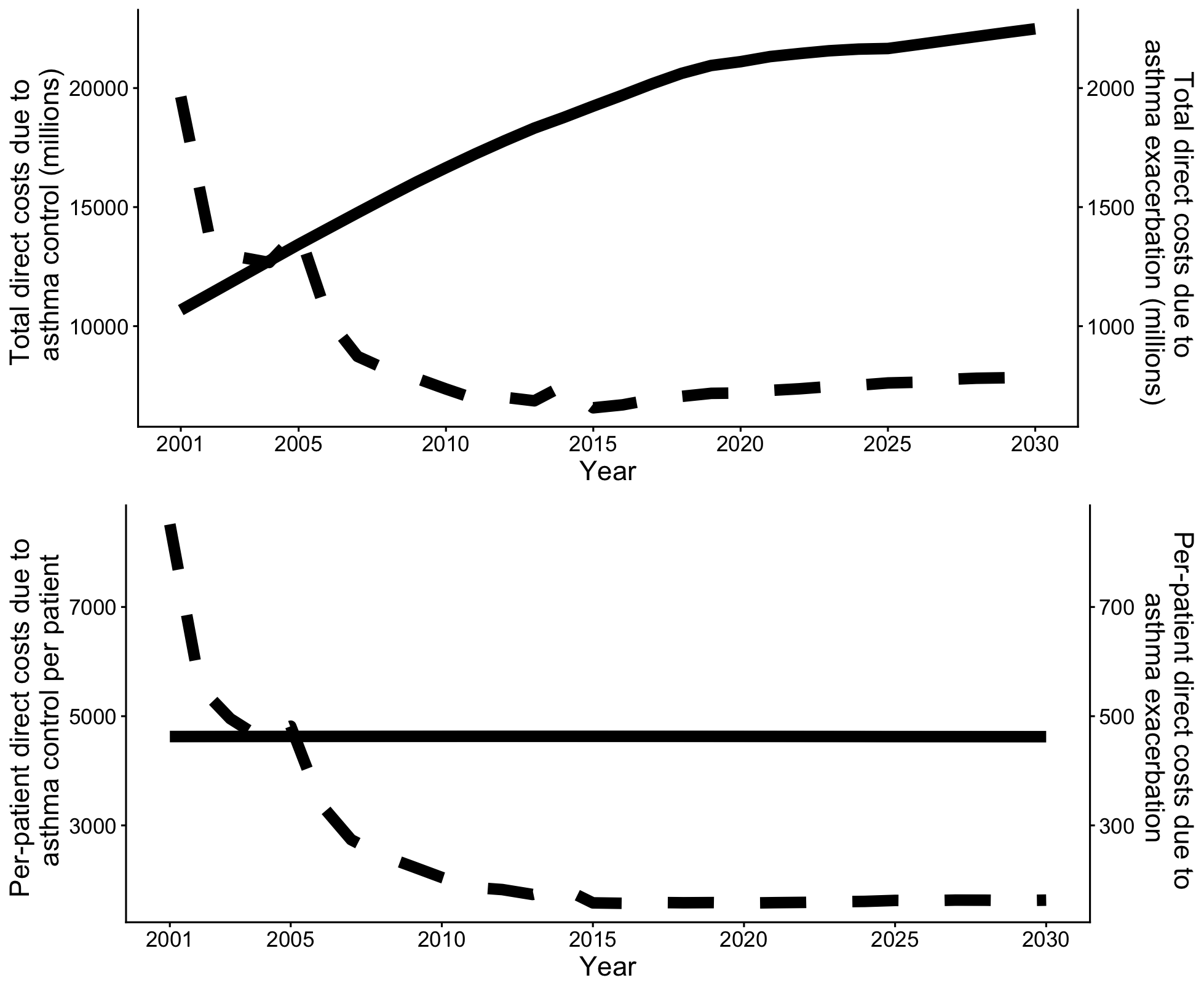
 **Supplementary Fig 10.** Simulated total direct costs (top) and per-patient direct costs in 2023 CAD due to asthma control (solid; left y-axis) and asthma exacerbation (dashed; right y-axis).


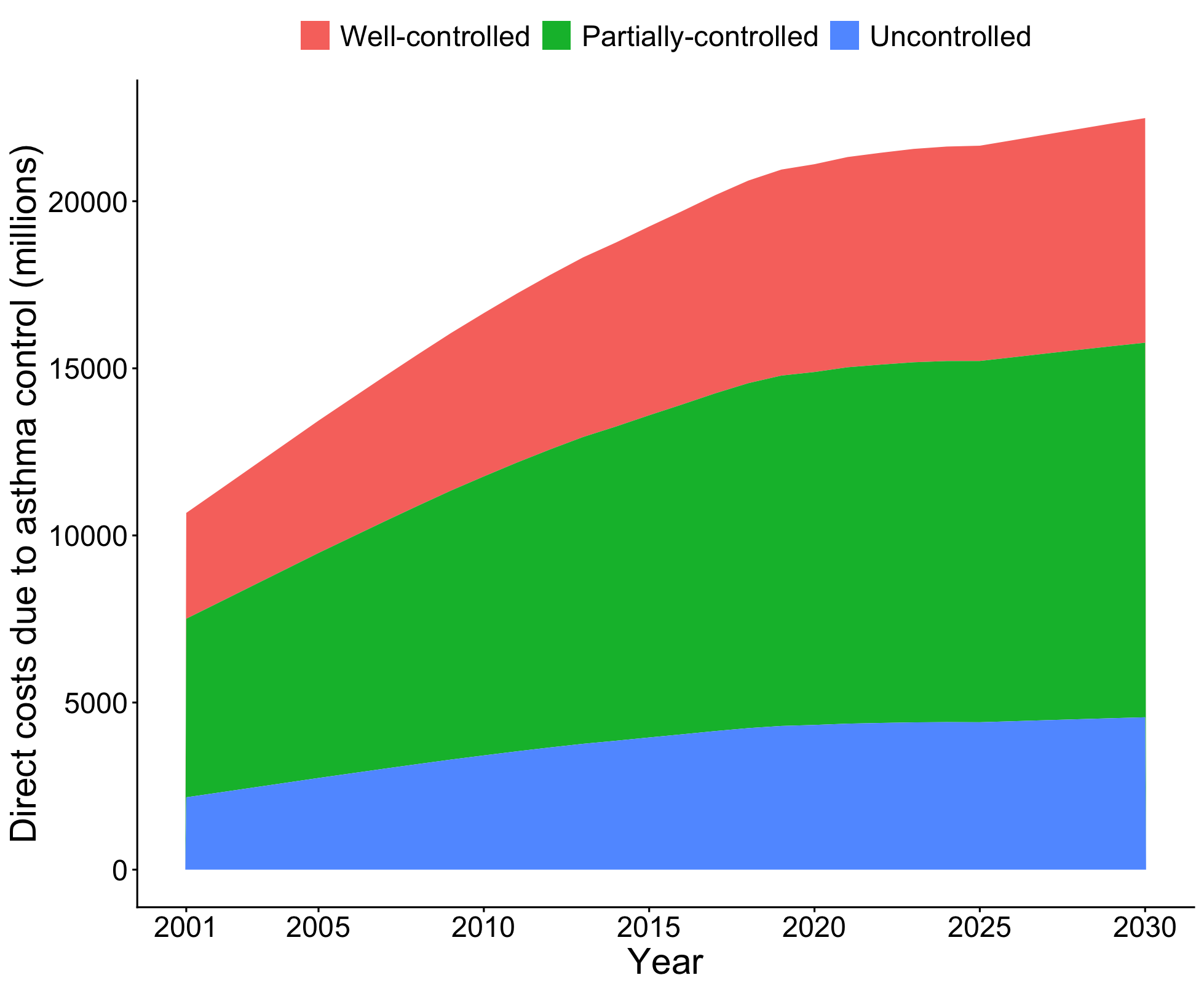


**Supplementary Fig 11.** Simulated direct costs due to asthma control by type.

**
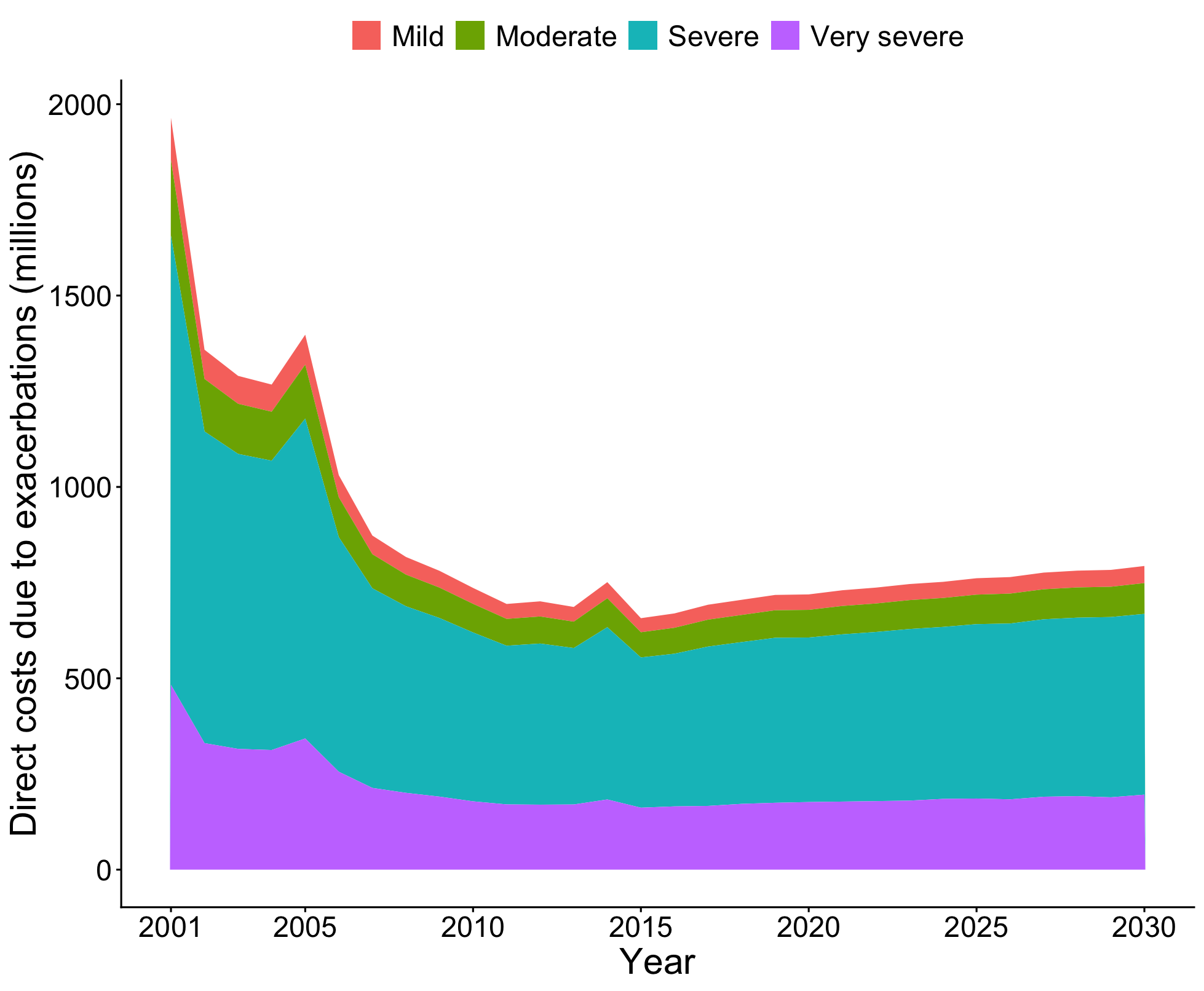
**

**Supplementary Fig 12.** Simulated direct costs due to asthma exacerbations by severity.

# References

1. Statistics Canada. Table 17-10-0005-01: Population estimates on July 1, by age and sex. 2022. Available: https://doi.org/10.25318/1710000501-eng

2. Statistics Canada. Table 17-10-0057-01: Projected population, by projection scenario, age and sex, as of July 1 (x 1,000). 2022. Available: https://doi.org/10.25318/1710005701-eng

3. Statistics Canada. Table 13-10-0837: Life Tables, Canada, provinces and territories. 2022. Available: https://doi.org/10.25318/1310083701-eng

4. O’Byrne P, Fabbri LM, Pavord ID, Papi A, Petruzzelli S, Lange P. Asthma progression and mortality: the role of inhaled corticosteroids. Eur Respir J. 2019;54: 1900491. doi:10.1183/13993003.00491-2019

5. Statistics Canada. Table 13-10-0394-01: Leading causes of death, total population, by age group. 2022. Available: https://doi.org/10.25318/1310039401-eng

6. Strauss D, Shavelle R, Brooks J. The life table. 2023. Available: https://www.lifeexpectancy.org/lifetable.shtml

7. Lee TY, Petkau J, Saatchi A, Marra F, Turvey SE, Lishman H, et al. Impact analysis of infant antibiotic exposure on the burden of asthma: a simulation modeling study. Front Allergy. 2024;5. doi:10.3389/falgy.2024.1491985

8. Statistics Canada. Canadian community health Survey—Annual component (CCHS). 2022.

9. British Columbia (BC) Ministry of Health. British Columbia Chronic Disease Registries (BCCDR) Case Definitions (Last Update: April 2022). 2022. Available: http://www.bccdc.ca/resource-gallery/Documents/Chronic-Disease-Dashboard/asthma.pdf

10. Canadian Institute for Health Information. CIHI data quality study of the 2005-2006 discharge abstract database. Canadian Institute for Health Information; 2009.

11. British Columbia Ministry of Health [creator]. Chronic Disease Dashboard. BC Observatory for Population and Public Health [publisher]; 2023. Available: http://www.bccdc.ca/health-info/disease-system-statistics/chronic-disease-dashboard

12. Global Initiative for Asthma. Global strategy for asthma management and prevention. Updated 2023. 2023. Available: https://www.ginasthma.org/reports

13. Thomas D, McDonald VM, Pavord ID, Gibson PG. Asthma remission: what is it and how can it be achieved? Eur Respir J. 2022;60: 2102583. doi:10.1183/13993003.02583-2021

14. Bisgaard H, Bønnelykke K. Long-term studies of the natural history of asthma in childhood. J Allergy Clin Immunol. 2010;126: 187–197. doi:10.1016/j.jaci.2010.07.011

15. Kavanagh J, Jackson DJ, Kent BD. Over- and under-diagnosis in asthma. Breathe. 2019;15: e20–e27. doi:10.1183/20734735.0362-2018

16. Patrick DM, Sbihi H, Dai DLY, Al Mamun A, Rasali D, Rose C, et al. Decreasing antibiotic use, the gut microbiota, and asthma incidence in children: evidence from population-based and prospective cohort studies. Lancet Respir Med. 2020;8: 1094–1105. doi:10.1016/S2213-2600(20)30052-7

17. Duong QA, Pittet LF, Curtis N, Zimmermann P. Antibiotic exposure and adverse long-term health outcomes in children: A systematic review and meta-analysis. J Infect. 2022;85: 213–300. doi:10.1016/j.jinf.2022.01.005

18. Hoskinson C, Dai DLY, Del Bel KL, Becker AB, Moraes TJ, Mandhane PJ, et al. Delayed gut microbiota maturation in the first year of life is a hallmark of pediatric allergic disease. Nat Commun. 2023;14: 4785. doi:10.1038/s41467-023-40336-4

19. Bonett DG. Transforming odds ratios into correlations for meta-analytic research. Am Psychol. 2007;62: 254–255. doi:10.1037/0003-066X.62.3.254

20. Chen W, Fitzgerald JM, Rousseau R, Lynd LD, Tan WC, Sadatsafavi M. Complementary and alternative asthma treatments and their association with asthma control: a population-based study. BMJ Open. 2013;3: e003360. doi:10.1136/bmjopen-2013-003360

21. Bateman ED, Boushey HA, Bousquet J, Busse WW, Clark TJH, Pauwels RA, et al. Can guideline-defined asthma control be achieved? The Gaining Optimal Asthma ControL study. Am J Respir Crit Care Med. 2004;170: 836–844. doi:10.1164/rccm.200401-033OC

22. Fuhlbrigge A, Peden D, Apter AJ, Boushey HA, Camargo C, Gern J, et al. Asthma Outcomes: Exacerbations. J Allergy Clin Immunol. 2012;129: S34–S48. doi:10.1016/j.jaci.2011.12.983

23. Bateman ED, Reddel HK, O’Byrne PM, Barnes PJ, Zhong N, Keen C, et al. As-Needed Budesonide–Formoterol versus Maintenance Budesonide in Mild Asthma. N Engl J Med. 2018;378: 1877–1887. doi:10.1056/NEJMoa1715275

24. Yaghoubi M, Adibi A, Zafari Z, FitzGerald JM, Aaron SD, Johnson KM, et al. Cost-effectiveness of implementing objective diagnostic verification of asthma in the United States. J Allergy Clin Immunol. 2020;145: 1367-1377.e4. doi:10.1016/j.jaci.2019.11.038

25. Lee TY, Petkau J, Sadatsafavi M. Long-Term Natural History of Severe Asthma Exacerbations and Their Impact on the Disease Course. Ann Am Thorac Soc. 2022;19: 907–915. doi:10.1513/AnnalsATS.202012-1562OC

26. Lee TY, Petkau J, Mangat N, Safari A, Cragg JJ, Lynd LD, et al. 16-year trends in asthma hospital admissions in Canada. Ann Allergy Asthma Immunol. 2022;129: 475-480.e2. doi:10.1016/j.anai.2022.06.022

27. Bezanson J, Edelman A, Karpinski S, Shah VB. Julia: a Fresh approach to numerical computing. SIAM Rev. 2017;59: 65–98. doi:10.1137/141000671
