# Supplementary material for: Development of an asthma policy model for Canada: Lifetime Exposures and Asthma outcomes Projection
Source: PLoS One. 2026 May 13;21(5):e0348878. doi: 10.1371/journal.pone.0348878 (PMC13170856; doi:10.1371/journal.pone.0348878)
Supplement: Attachment — Submitted filename: response_document.docx [file pone.0348878.s003.docx]

Reviewers' comments:

Reviewer's Responses to Questions

**Comments to the Author**

1. Is the manuscript technically sound, and do the data support the conclusions?

The manuscript must describe a technically sound piece of scientific research with data that supports the conclusions. Experiments must have been conducted rigorously, with appropriate controls, replication, and sample sizes. The conclusions must be drawn appropriately based on the data presented.

Reviewer #1: Yes

Reviewer #2: Yes

Reviewer #3: Yes

2. Has the statistical analysis been performed appropriately and rigorously?

Reviewer #1: No

Reviewer #2: Yes

Reviewer #3: Yes

3. Have the authors made all data underlying the findings in their manuscript fully available?

The [PLOS Data policy](https://urldefense.com/v3/__http:/track.editorialmanager.com/CL0/http:*2F*2Fwww.plosone.org*2Fstatic*2Fpolicies.action*23sharing/1/010f019ba18bcb79-c13861c3-5d40-4620-a771-432c4145ae23-000000/9LyZihBN3kUCRzq_4KUTtNvj5E9DNNYVwrihVyUxBqs=245__;JSUlJSU!!G92We9drHetJ8EofZw!fwsJlOWc49pR-thXcXxQUTy5WfjbwFrNe6eb4tsmnSkW4K_qu8eNoneTLF-bD3EYqXuSKp15b6yqG-OmD3Ew$) requires authors to make all data underlying the findings described in their manuscript fully available without restriction, with rare exception (please refer to the Data Availability Statement in the manuscript PDF file). The data should be provided as part of the manuscript or its supporting information, or deposited to a public repository. For example, in addition to summary statistics, the data points behind means, medians and variance measures should be available. If there are restrictions on publicly sharing data—e.g. participant privacy or use of data from a third party—those must be specified.

Reviewer #1: Yes

Reviewer #2: Yes

Reviewer #3: No

4. Is the manuscript presented in an intelligible fashion and written in standard English?

PLOS ONE does not copyedit accepted manuscripts, so the language in submitted articles must be clear, correct, and unambiguous. Any typographical or grammatical errors should be corrected at revision, so please note any specific errors here.

Reviewer #1: Yes

Reviewer #2: Yes

Reviewer #3: Yes

5. Review Comments to the Author

Please use the space provided to explain your answers to the questions above. You may also include additional comments for the author, including concerns about dual publication, research ethics, or publication ethics. (Please upload your review as an attachment if it exceeds 20,000 characters)

**Reviewer #1:**

**RESPONSE:** Dear Reviewer 1, thank you for your insightful comments. Below please find our point-by-point responses (black) to your comments (greyed-out). We hope that the revisions and clarifications provided also address your concerns.

Line numbers in this response document refer to those in the clean revised manuscript, not the version tracked changes.

1. Lines 419–421: The authors state, “The current version of the asthma model does not account for uncertainty in parameters... Our conscious choice was to initially emphasise proper modelling of the structure.” Can the authors justify publishing a policy model that cannot currently generate confidence intervals or credible intervals for its projections? When will the parameter distributions be specified?

**RESPONSE:** We appreciate this critical point. Our decision to prioritize the model structure was based on the necessity of establishing a framework for developing a reference model, which requires significant resources and coordinated efforts with stakeholders. We acknowledge that the absence of parameter uncertainty quantification is a key limitation – as noted in the Discussion. However, there is a clear precedent for the value of publishing structural frameworks and projections without accompanying CIs. For example, several CISNET lung cancer models, which have informed national lung cancer screening guidelines in the U.S., do not account for parameter uncertainty (<https://www.uspreventiveservicestaskforce.org/uspstf/document/modeling-study/lung-cancer-screening>). While we believe that the current version provides the necessary foundation for the asthma research community, we fully support the need to incorporate parameter uncertainty quantification. Of note, we account for stochastic uncertainty; see the Monte Carlo error provided an applied study using the LEAP model (PMID: 39764437; see a snapshot of Table 2 in that study).

It is in our agenda to embed a probabilistic framework into the LEAP model so that parameter uncertainty can be fully propagated to model outputs in the form of CIs. Once the core structure of the model is published, we will have much room in a dedicated publication to fully outline our approach for creating a probabilistic parameter space that captures our current knowledge. This is a complex task as several model parameters are estimated via calibration techniques that require detailed explanations.

2. Lines 201–204: The model simulates “labelled (diagnosed)” asthma rather than “true” physiological asthma. How does the model differentiate between an intervention that reduces disease onset versus an intervention that simply reduces diagnosis rates? If true asthma states are not modeled, how can the model evaluate screening or case-detection policies effectively?

**RESPONSE:** We appreciate this important distinction. Modeling the true (latent) state of asthma separately from the labeled state is challenging, because unlike conditions with definitive biomarkers like COPD or certain cancers, asthma lacks a gold-standard diagnostic test, particularly in pediatric populations. In fact, the very definition of asthma at the physiological level is the subject of much debate – this is different, for example, from cancers or diabetes. Without clear consensus and robust data, attempting to model a complex physiological system with a single status (presence or absence of underlying asthma) might limit the credibility and applicability of this model.

As such, we chose to model labeled asthma to remain grounded in observable data. We do not consider this as a methodological drawback – rather as reflecting the nature of the condition and the available evidence. Our model structure accommodates modeling asthma diagnosis, as well as asthma-related events such as exacerbations, will be governed by risk equations. Please note that even if an intervention reduces asthma diagnosis rates, the risk equations for symptoms and exacerbations will still operate. This will be different from the effect of an intervention that reduces the risk of asthma symptoms and exacerbations – in turn reducing diagnosis rate.

However, before such risk questions are fully developed, the model will not be able to inform screening / case detection strategies. We highlight this issue in the Discussion section (Lines 441–448):

*“Currently, the natural course of asthma is modelled through labelled/diagnosed, rather than true, asthma state based on objective measures of reversible airflow obstruction and airway hyperresponsiveness. This was deemed sufficient for the first set of policy questions LEAP is intended to tackle, which are about preventive strategies that reduce the risk of future asthma. The outcomes of interest related to such policies are future asthma cases and their related outcomes. To address some other policy questions, such as the benefit of screening, case detection, and case verification policies, true asthma states should be modelled in the future upon accumulation of sufficient evidence on true asthma states.”*

3. Lines 184–186: The model currently only includes Age, Sex, Family History, and Antibiotic Exposure. Given the stated objective to support “health policy planning” (Line 31), is the inclusion of only one modifiable risk factor (antibiotics) sufficient to validate the model structure? Why were strong predictors like prematurity or maternal smoking excluded from the first version?

**RESPONSE:** We would like to clarify that the current study is focused on the validation of core structural components of the model. The selection of Age, Sex, Family History, and Antibiotic Exposure was a deliberate choice for the initial version of the LEAP model, in consultation with the steering committee, based on robust evidence at hand. As noted in the Discussion, incorporating additional key risk factors, such as maternal smoking and exposure to outdoor air pollution, is a primary need to allow for evaluation of a wide range of emerging interventions (Lines 472–479):

*“To identify priority areas, we have held a series of discussion sessions with the 8 stakeholders representing clinical (pediatric and adult respirologist), government (Health Canada), non-profit (BC Lung Foundation), and patient perspectives.[59] We are actively expanding the LEAP model (e.g., incorporating additional risk factors such as smoking and outdoor air pollution exposure and indirect costs) and plan to address the following priority areas with buy-in from stakeholders: outdoor air pollution and cost-effectiveness of a portable HEPA air cleaner distribution program, expanded access to smoking cessation programs, and respiratory syncytial virus vaccine for prevention of early childhood infection”*

We anticipate that this will require specific structural refinements (e.g., changing the cycle unit to daily to model the impact of wildfire smoke). Our model is built from the grounds up, both structurally and in its implementation (programmatic choices) to accommodate such growth and flexibility. Ultimately, we have designed the LEAP model as a living platform: an open-source, modular framework intended for the research community to collaboratively expand and refine over time.

4. Lines 400–403: The authors extrapolate British Columbia (BC) incidence/prevalence data to the entire Canadian population, justifying it by minimal difference in CCHS data. Canada has significant regional heterogeneity in asthma rates (e.g., Atlantic Canada often shows higher rates than the West). Relying on BC administrative data (which depends on BC-specific billing codes and physician behaviours) risks biasing the national model. Did the authors perform a sensitivity analysis using provincial adjusters based on CCHS data to see if regional variations alter the aggregate national outcomes?

**RESPONSE:** We appreciate this insight regarding regional heterogeneity. We acknowledge that relying on the BC administrative data alone may not fully capture the nuances of physician billing behaviors or higher asthma prevalence that differ across regions. We have updated the Discussion to explicitly acknowledge this as a limitation (Lines 414–418):

*“Nevertheless, relying on a single province may not fully capture regional heterogeneity in clinical practices or disease trajectories, potentially limiting geographic generalisability of the model to other provinces or territories. It would be more desirable to estimate incidence and prevalence rates from a sample representative of the entire country or explicitly differentiate asthma occurrence and outcomes between provinces and territories.[48,49]”*

We are currently exploring strategic partnerships with researchers in other provinces to explicitly model regional variations in future versions of the LEAP model.

5. Line 140: The model uses an annual time cycle. Asthma exacerbations are acute events lasting days. Infants undergo rapid developmental changes. Is an annual cycle granular enough to capture the immediate effects of infant antibiotic exposure (which occurs in months 0-12) or the seasonality of exacerbations?

**RESPONSE:** Thanks for the insight. Please note that while the time cycle is one year, ‘snapshot’ events – events with much shorter durations than the time cycle – can be modeled as counts during a single time cycle. As such, LEAP can model exacerbations. However, we agree that some aspects of exacerbation history will be lost if they are modeled as individual-level annual counts. For example, we might not be able to evaluate the impact of early intervention at the outset of an exacerbation.

However, we would like to emphasize that the choice of annual cycles is for the current implementation of the model, and the model structure is created such that the user can change the cycle duration. We are currently updating the LEAP model to support other temporal resolutions in a subsequent version, starting with the monthly cycle. This will allow researchers to choose the most appropriate unit cycle for their intervention of interest. It will also be possible to temporarily increase the time granularity (e.g., switching to daily time cycle for the duration of an event, and then jumping to the beginning of the next cycle).

An annual cycle seems appropriate for modeling the effects of infant antibiotic exposure on asthma incidence, as this relationship reflects a long-term development trajectory. This choice thus reflects the current state of LEAP and the questions it is addresses, while not being a fundamental limitation.

6. Line 191: The model assumes all individuals have a 29.3% probability of family history, regardless of birth year. Since asthma prevalence has increased over the last 30 years (according to the Introduction), shouldn't the rate of parental history also be increasing for later cohorts? A child born in 2020 is more likely to have asthmatic parents than a child born in 1980.

**RESPONSE:** Thank you for your comment. Kindly note that we did not mention that asthma prevalence has increased over the last 30 years in the Introduction. Asthma prevalence has been stable in Canada in the last 10 years or so based on national annual survey data (from 2015 to 2022). However, asthma prevalence did increase for several provinces in an earlier period, as shown by a study in Alberta from 1995 to 2015 (PMID: 33061999), suggesting potential cohort effects that should be considered in the future. We have added this in the Discussion (Lines 418–424):

*“Based on the stable trends in asthma prevalence reported in national annual surveys from 2015 to 2022,[36] we assumed a constant prevalence for family history of asthma across all simulated individuals. However, as some Canadian provinces have reported increasing trends in asthma prevalence,[50] this assumption may lead to a misestimation of family history prevalence in those regions. Future work should incorporate regional heterogeneity and account for potential cohort effects on the parameters.”*

7. Line 220: The authors introduced asthma reassessment to calibrate prevalence. Is this mechanism structurally equivalent to remission? Does an individual who loses the asthma label return to the “susceptible” pool and become at risk for ‘incidence’ again, or are they immune? The handling of ‘re-diagnosis’ is crucial for longitudinal accuracy.

**RESPONSE:** Correct, this reassessment mechanism is structurally equivalent to remission. Within the model, individuals who lose the asthma label are returned to the “susceptible” pool and are once again at risk for asthma incidence. Indeed, in current practice, asthma remission is also mostly defined based on the long-term absence of symptoms and exacerbations (again, referring to the lack of a definite marker for asthma activity).

This is discussed further down in Section 3.1, but we have expanded on that line for clarification (Lines 221-224):

*“Then we introduced asthma reassessment to calibrate the age- and sex-specific crude incidence and prevalence for each year. This mechanism is equivalent to remission, wherein an individual previously labelled with asthma can become non-asthmatic, return to the susceptible pool, and be at risk of asthma incidence in subsequent cycles.”*

8. Line 266: The rates (0.188 for WC, 0.564 for UC) seem to be derived from the GOAL study (2004) and EBA study. Treatment paradigms have shifted significantly since 2004 (e.g., widespread use of biologicals, SMART therapy). Are these exacerbation rates representative of current Canadian clinical practice?

**RESPONSE:** Correct. The rates were derived by combining evidence from the GOAL (USA) and EBA (Canada) studies, and the severe exacerbation rates were calibrated using the recent national Canadian hospitalization data. However, the distribution of exacerbation severity levels may not fully reflect contemporary practice given the recent temporal shifts in treatment paradigms as you noted. This limitation is now discussed in the revised manuscript (Lines 425–430):

*“We calibrated severe exacerbation rates against observed values in the national Canadian hospitalization database. However, this calibration does not guarantee that the rates for other severity levels align with current practice. This discrepancy arises because the baseline distribution of severity levels was derived from cohort studies, which may not fully represent the Canadian population or reflect shifts in treatment paradigms, such as widespread use of biologics.”*

9. Line 278: The model assumes utility starts at 1 (perfect health) at birth. This assumption is contrary to most HTA guidelines, as infants often have temporary health states (colic, infections) that reduce utility below 1.0. Does this assumption skew QALY calculations for early-childhood interventions?
 **RESPONSE:** We agree that this assumption could potentially skew QALY calculations for interventions that affect age < 6 years. This interpolation appears to closely match the utility values for youth (6-11 years and 7-12 years old) reported in a different Canadian study albeit they used a different tool (HUI3). We have expanded our discussion to address this issue (Lines 431–440):

*“Furthermore, we extrapolated health state utility values for children under 18 years of age under the assumption of perfect health at birth. While this interpolation matched the utility values reported for children aged 6 years or older in other Canadian studies using the Health Utility Index Mark 3,[51] it may not well represent the utility values for those under 6 years of age, as the assumption of perfect health at birth may be considered overly optimistic. We suggest sensitivity analyses utilising lower utility values for this younger age group. In addition, we could not incorporate caregiver or family utility effects due to lack of evidence in Canada. The current version of the LEAP model did not incorporate indirect costs, such as productivity losses from missed school days for children and work absenteeism for adults, which represent a substantial proportion of the economic burden of asthma.[52,53]”*

10. Line 354: ‘The simulated rate of very severe exacerbations was slightly higher than the target value.’ How does this overestimation impact the cost analysis, given that very severe exacerbations (hospitalizations) are likely the primary cost drivers?

**RESPONSE:** While very severe exacerbations are very costly, the impact of this slight overestimation on the overall cost is not substantial. As shown in Supplementary Figures 11 and 12 (as per suggestion by Reviewer 3), the direct costs associated with maintenance therapy and day-to-day asthma control constitute a major portion of the total direct costs, which helps buffer the model against this minor deviation. Nonetheless, this deviation should be addressed in future updates with a better calibration technique; this point has been addressed in the Discussion (Lines 398-400):

*“Moreover, we observed a slight overestimation of severe exacerbation rates, particularly among males. To minimise these discrepancies, more granular calibration techniques could be employed, such as the use of deep learning based Bayesian calibration.[47]”*

11. Line 115: “June 31, 2025”. June has only 30 days.

**RESPONSE:** Thanks for catching this error; it has been fixed.

12. Line 164 (Table 1 Footnote): Please ensure mathematical notation is consistent throughout the table and text.

**RESPONSE:** Thanks, we have checked and believe that the notation is consistent.

13. Line 210: “Asthma was not modelled for children under 3 years of age”. How does this interact with the ‘Infant Antibiotic Exposure’ module? If exposure happens at age <1, but asthma "starts" at age 3, is the risk factor stored as an attribute until the agent turns 3?

**RESPONSE:** Yes, all the relevant risk factors and events are stored for each agent. This is feasible because we assume no interactions among agents, allowing us to run each agent at a time.

14. Line 296 (Inflation): “1 USD in 2018 = 1.22 USD in 2023”. This implies a cumulative inflation of 22% over 5 years. While inflation was high recently, please cite the specific CPI calculator or source used for this conversion.

**RESPONSE:** Thanks, the specific CPI calculator has been cited in the revision version (we used <https://www.bls.gov/data/inflation_calculator.htm>).

AFTER A DEEP REVIEW I FOUND SOME QUESTIONS MENTIONED HERE ABOVE AND MISTAKES AND ERRORS. I request to take care of these.

**RESPONSE:** Thank you for your thorough review and valuable comments.

**Reviewer #2:**

I am pleased to have the opportunity to review this manuscript. This study presents the methodology of the LEAP model, developed to support asthma prevention and policy-oriented decision-making, and represents an important contribution as a national-level, open-source policy model. Overall, the manuscript is well designed and presents an interesting and timely study.

**RESPONSE:** We would like to thank the reviewer for their positive assessment of our work. We appreciate the reviewer’s thoughtful comments and suggestions, which have helped us improve the clarity and depth of the manuscript. We have addressed each of the specific comments below and have incorporated the corresponding changes into the revised version of the paper.

Line numbers in this response document refer to those in the clean revised manuscript, not the version tracked changes.

1) The LEAP model is designed and calibrated for a specific population—namely, the Canadian population—within the context of a particular healthcare system. As such, conducting external validation in the conventional sense may be inherently challenging due to these structural constraints. Nevertheless, to enhance the generalizability and credibility of the model, a more detailed discussion of potential strategies for external validation—such as validation across different regions, time periods, or independent data sources—would be valuable in future work.

**RESPONSE:** We concur. To address this, we have expanded the Discussion to emphasize the importance of forming strategic partnerships with international researchers. These collaborations will facilitate dedicated external validation studies using independent regional datasets and longitudinal cohorts. We believe these steps are essential to establishing the generalizability and long-term credibility of the LEAP model across diverse clinical and geographic contexts (Lines 454–460):

*“Further, future efforts should focus on dedicated external validation studies to assess the generalisability of the LEAP model across different populations and time periods. This includes temporal validation to ensure the model structure remains robust across time as well as to geographic validation through strategic collaborations both locally (e.g., using the pan-Canadian respiratory electronic health records[56]) and internationally (e.g., via the Copenhagen Prospective Studies on Asthma in Childhood[57] and the International Severe Asthma Registry[58]).”*

2) At present, the model incorporates a relatively limited set of risk factors, primarily focusing on basic demographic characteristics and selected early-life exposures. Nevertheless, given the stated goal of supporting population-level policy evaluation, further incorporation of additional environmental, behavioral, and clinical risk factors would substantially enhance the model’s applicability and policy relevance.

**RESPONSE:** We fully agree that broadening the scope of risk factors is essential for the model’s long-term policy relevance. As noted in the Discussion, we are currently planning to incorporate additional risk factors in consultation with patient partners and other interest holders. This collaborative process aims to prioritize environmental, behavioral, and clinical factors that will enable the LEAP model to evaluate a wider array of public health and precision medicine interventions. This is the main goal of creating the structure of a flexible, upgradable reference policy model.

3) The selection of risk factors appears to be grounded in a previously published concept map developed through a modified Delphi process. However, the detailed procedures of this consensus-building process are not fully described in the current manuscript. Given that this step is foundational to the construction of the model, greater transparency regarding the Delphi process is critical. In addition, it would be helpful to clarify whether future updates and expansions of the model will follow the same methodological framework.

**RESPONSE:** We appreciate the reviewer’s emphasis on the importance of the Delphi process. While we agree that this foundation is critical, for details we refer readers to the previously published study dedicated to the concept map to avoid significant overlap and keep the current manuscript focused on the model structure and validation: <https://bmjopenrespres.bmj.com/content/8/1/e000881>

Regarding future updates, we are actively consulting with patient partners and stakeholders to guide the model's evolution. We have updated the Discussion to include a reference to our recently published work on patient and public involvement within the LEAP model, which outlines the methodological framework for these ongoing expansions (PMID: 41269490; Lines 472–474):

*“To identify priority areas, we have held a series of discussion sessions with the 8 stakeholders representing clinical (pediatric and adult respirologist), government (Health Canada), non-profit (BC Lung Foundation), and patient perspectives.[59]”*

4) Many parameters used in the long-term projections appear to be derived from short- to medium-term data and are treated as time-invariant. Incorporating parameter uncertainty, particularly regarding the temporal stability of these estimates, would strengthen the robustness of the model’s long-term projections.

**RESPONSE:** We agree and acknowledge that the absence of uncertainty quantification is a key limitation – as noted in the Discussion. However, there is a clear precedent for the value of publishing structural frameworks and projections without accompanying CIs. For example, several CISNET lung cancer models, which have informed national lung cancer screening guidelines in the U.S., do not account for parameter uncertainty (<https://www.uspreventiveservicestaskforce.org/uspstf/document/modeling-study/lung-cancer-screening>). While we believe that the current version provides the necessary foundation for the asthma research community, we fully support the need to incorporate uncertainty quantification.

Importantly, we are planning to embed a probabilistic framework into the LEAP model so that parameter uncertainty can be fully propagated to model outputs in the form of CIs. Once the core structure of the model is published, we will have much room in a dedicated publication to fully outline our approach for creating a probabilistic parameter space that captures our current knowledge. This is a complex task as several model parameters are estimated via calibration techniques that require detailed explanations. To address your concern, we have added the importance of temporal validation in the revision (see our response to your first comment).

5) As a minor point, the resolution of several figures appears to be low. Providing higher-resolution figures would improve the clarity and readability of the manuscript.

**RESPONSE:** Thanks, we will provide higher-resolution figures for a publication version.

**Reviewer #3:**

Thank you for this important modelling work. My comments are minor:

**RESPONSE:** Dear Reviewer 3, we are grateful for your comments. Of note, regarding the availability of data for this study, we made all codes and output from the simulation available via the GitHub repository: <https://github.com/tyhlee/LEAP.jl>. The publicly available input datasets are also available, but a couple of cohort datasets are not available due to privacy, which we stated under the ‘Availability of data and materials’ section in the title page in the original submission as follows:

*“The datasets generated and/or analysed during the current study are available in the GitHub repository (*[*https://github.com/tyhlee/LEAP.jl*](https://github.com/tyhlee/LEAP.jl)*). Restricted datasets that support the findings of this study are the Chronic Disease Registry (*[*https://assets-hdpbc.healthbc.org/doc/show/hdp/d06baaea-6692-4118-90ca-7de0a85177ea*](https://assets-hdpbc.healthbc.org/doc/show/hdp/d06baaea-6692-4118-90ca-7de0a85177ea)*) from the Ministry Health of British Columbia (complete the following instructions here to gain access:* [*https://www2.gov.bc.ca/assets/gov/health/forms/5456fil.pdf*](https://www2.gov.bc.ca/assets/gov/health/forms/5456fil.pdf)*) and the Canadian Healthy Infant Longitudinal Development Study Data (*[*https://childcohort.ca/for-researchers/data-access/*](https://childcohort.ca/for-researchers/data-access/)*), but restrictions apply to the availability of these data, which were used under license for the current study, and so are not publicly available. Please refer to the instructions in the URLs provided to obtain access to the restricted datasets.”*

Line numbers in this response document refer to those in the clean revised manuscript, not the version tracked changes.

Abstract
- Under results, it was stated that "Age-sex stratified demographic projections from the model closely matched projections". Does the second “projection” refer to those published from national surveys?

**RESPONSE:** The demographic projections refer to those published by Statistics Canada: <https://www150.statcan.gc.ca/n1/pub/17-20-0003/172000032026001-eng.htm> (of note, this is a newer version that has been updated early this year 2026). Newer projection data will be incorporated into the next version of the LEAP model (work in progress). We have added this information to the revision (Lines 389–391):

*“Of note, Statistics Canada has recently released detailed migration data, which will allow for the modelling of actual numbers in the next version.[46]”*

Methods
- May I suggest putting key equations such as Equation 2 from the supplementary material back to the main text?

**RESPONSE:**  We appreciate this suggestion and gave it careful consideration. However, we have decided to keep the primary technical equations in the Supplementary Materials. This conscious decision was made in consultation with the steering committee to ensure that the main manuscript remains accessible to a broad, non-technical audience (clinicians, policy-makers).

- Line 190: “We found that 29.3% (95% CI: 27.3%,31.1%) of the children in the study had parental asthma.” Were these estimates derived by the authors from the cited study?

**RESPONSE:** Yes, we used data from the CHILD study to obtain these estimates.

- Is there a reason why published transition probabilities were not used to model the progression of asthma across the control states?

**RESPONSE:** We decided to use the data from a Canadian study (the Economic Burden of Asthma study) to model the transition probabilities across the control states, as there is substantial between-country heterogeneity in asthma control levels (PMID: 15241342).

Result
- Line 363 - Does the decline in severe and very severe exacerbations overtime algin with expectations

**RESPONSE:** Yes, this decline is consistent with observed trends. National data show a decreasing trend in asthma-related hospitalizations, likely reflecting improvements in contemporary asthma management and the increased efficacy of maintenance therapies in preventing severe and very-severe exacerbations.

Discussion
- Line 405 – what does XXX represent?

**RESPONSE:** Thank you for pointing out this placeholder; this has been addressed in the revised version as (Lines 425–30):

*“We calibrated severe exacerbation rates against observed values in the national Canadian hospitalization database. However, this calibration does not guarantee that the rates for other severity levels align with current practice. This discrepancy arises because the baseline distribution of severity levels was derived from cohort studies, which may not fully represent the Canadian population or reflect shifts in treatment paradigms, such as widespread use of biologics.”*

- Is the inclusion of indirect cost into LEAP a potential direction of future work?
 **RESPONSE:** Yes – we are planning to incorporate indirect cost in the next version; this has been addressed in the Discussion:

At Line 437:

*“The current version of the LEAP model did not incorporate indirect costs, such as productivity losses from missed school days for children and work absenteeism for adults, which represent a substantial proportion of the economic burden of asthma.[52,53]”*

At Line 474:

*“We are actively expanding the LEAP model (e.g., incorporating additional risk factors such as smoking and outdoor air pollution exposure and indirect costs) …”*

Supplementary Material
- May I suggest adding a table of content with links to the different sections/pages.

**RESPONSE:** Thank you for this suggestion; a table of content with links has been added.

- Page 9 of supplementary material - in line 2, "crude asthma and incidence"

**RESPONSE:** Thanks for catching this omission; this has been changed to: crude asthma prevalence and incidence.

- Since direct cost is an outcome of interest, could the authors detail its components and corresponding estimates?

**RESPONSE:** Thank you for the suggestion; we have added two supplementary figures to show the direct cost due to control and exacerbation by components; please see Supplementary Figures 11 and 12, respectively.

6. PLOS authors have the option to publish the peer review history of their article ([what does this mean?](https://urldefense.com/v3/__https:/track.editorialmanager.com/CL0/https:*2F*2Fjournals.plos.org*2Fplosone*2Fs*2Feditorial-and-peer-review-process*23loc-peer-review-history/1/010f019ba18bcb79-c13861c3-5d40-4620-a771-432c4145ae23-000000/22wBKgJzPRvCwO-pUK7uf5CRh76Wh6kC3JBwqoPSD40=245__;JSUlJSUl!!G92We9drHetJ8EofZw!fwsJlOWc49pR-thXcXxQUTy5WfjbwFrNe6eb4tsmnSkW4K_qu8eNoneTLF-bD3EYqXuSKp15b6yqG4LCnia0$)). If published, this will include your full peer review and any attached files.

If you choose “no”, your identity will remain anonymous but your review may still be made public.

**Do you want your identity to be public for this peer review?** For information about this choice, including consent withdrawal, please see our [Privacy Policy](https://urldefense.com/v3/__https:/track.editorialmanager.com/CL0/https:*2F*2Fwww.plos.org*2Fprivacy-policy/1/010f019ba18bcb79-c13861c3-5d40-4620-a771-432c4145ae23-000000/kDiYMz0PQkAE281nVbby0DoA1bLTQm15IP5JpxtUeP4=245__;JSUl!!G92We9drHetJ8EofZw!fwsJlOWc49pR-thXcXxQUTy5WfjbwFrNe6eb4tsmnSkW4K_qu8eNoneTLF-bD3EYqXuSKp15b6yqGwhdX7k3$).

Reviewer #1: **Yes:** sri chakradhar challagali

Reviewer #2: No

Reviewer #3: No
